# Clinical Features and Outcomes of 23 Patients with Wiskott-Aldrich Syndrome: A Single-Center Experience

**DOI:** 10.4274/tjh.galenos.2020.2020.0334

**Published:** 2020-11-19

**Authors:** Şule Haskoloğlu, Ayşenur Öztürk, Gökcan Öztürk, Sevgi Kostel Bal, Candan İslamoğlu, Kübra Baskın, Serdar Ceylaner, Lale Tufan Satıroğlu, Figen Doğu, Aydan İkincioğulları

**Affiliations:** 1 Ankara University School of Medicine, Department of Pediatrics, Division of Immunology and Allergy, Ankara, Turkey; 2 Ankara University School of Medicine, Department of Pediatrics, Division of Genetic Diseases, Ankara, Turkey; 3 Ankara University School of Medicine, Department of Pediatrics, Ankara, Turkey; 4 Intergen Genetic Diagnosis Center, Ankara, Turkey

**Keywords:** Wiskott-Aldrich syndrome, Hematopoietic stem cell transplantation, Microthrombocytopenia, Outcome

## Abstract

**Objective:**

Wiskott-Aldrich syndrome (WAS) is an X-linked primary immune deficiency characterized by microthrombocytopenia, eczema, and recurrent infections. We aimed to evaluate the clinical features and outcomes of a WAS cohort.

**Materials and Methods:**

We retrospectively evaluated the clinical courses, immunological features, treatments, and outcomes in a total of 23 WAS patients together with data related to 11 transplanted cases among them between 1982 and 2019.

**Results:**

Before admission, 11 patients (48%) were misdiagnosed with immune thrombocytopenia. WAS scores were mostly 4 or 5. Eleven patients were transplanted and they had an overall survival rate of 100% during a median follow-up period of 8.5 years (range: 8 months to 20 years). Five patients who were not transplanted died at a median of 7 years (range: 2-26 years). Nontransplanted patients had high morbidity due to organ damage, mostly caused by autoimmunity, bleeding, and infections. Two novel mutations were also defined.

**Conclusion:**

All male babies with microthrombocytopenia should be evaluated for WAS. Hematopoietic stem cell transplantation should be performed at the earliest age with the best possible donors.

## Abstract (tr)

**Amaç:**

Wiskott-Aldrich Sendromu (WAS) X’e bağlı geçen mikrotrombositopeni, egzema ve tekrarlayan enfeksiyonlarla karakterize bir primer immün yetmezliktir.

**Gereç ve Yöntemler:**

1982-2019 yılları arasında izlediğimiz toplam 23 WAS’li hastanın klinik seyirleri, immünolojik özellikleri ve nakil yapılan 11 hastanın nakil ilişkili verileri retrospektif olarak değerlendirildi.

**Bulgular:**

Başvurudan önce 11 hasta yanlışlıkla immün trombositopeni tanısı almıştı. WAS skoru çoğunlukla 4 ve 5 puandı. On bir hastaya nakil yapıldı ve ortanca 8,5 yıl (8 ay-20 yıl) izlem süresinde hayatta kalma oranı %100 oldu. Nakil yapılamayan 5 hasta ortanca 7 yılda (2-26 yıl) kaybedildi. Nakil yapılmayan hastalar çoğunlukla otoimmünite, kanama ve enfeksiyonların neden olduğu organ hasarları nedeniyle yüksek morbiditeye sahipti. Ayrıca, iki yeni mutasyon tanımlandı.

**Sonuç:**

Mikrotrombositopenisi olan tüm erkek bebekler WAS açısından değerlendirilmelidir. Hematopoetik kök hücre nakli mümkün olan en iyi donörlerle en erken yaşta yapılmalıdır.

## Introduction

Wiskott-Aldrich syndrome (WAS) is a rare X-linked primary immunodeficiency characterized by immunodeficiency, thrombocytopenia, and eczema [1,2]. The *WAS* gene encodes the WAS protein (WASp) [3]. WASp is an important regulator of the actin cytoskeleton required for many hematopoietic and immune cell functions [4,5]. Estimated incidence of WAS is one in 100,000 live male births [6]. The presence and severity of the clinical findings are variable [7]. The severity of the clinical presentation is measured by the WAS score described by Zhu et al. [8]. Patients with a WAS score of ≥3 points are regarded as having a severe phenotype [8,9].

The first patient with WAS in Turkey (P1 in this cohort) was diagnosed by Babacan et al. [10] in 1982. Currently, hematopoietic stem cell transplantation (HSCT) is the most important curative treatment for WAS [11,12,13,14,15,16,17]. We aimed to evaluate the clinical features and outcomes of our WAS cohort as a synopsis of a single-center experience with long-term follow-up.

## Materials and Methods

From 1982 to 2019, 23 WAS patients from 15 families were diagnosed and followed at the Ankara University Medical School’s Department of Pediatric Immunology and Allergy. WAS diagnosis was confirmed according to the criteria of the European Society of Immune Deficiency [18]. Parental consent was obtained in all cases. The WAS score, whereby each clinical feature is given 1 point, was used to evaluate the clinical severity of the patients. Patients with microthrombocytopenia without any other clinical or laboratory signs received a score of 1. Patients with platelet abnormalities and moderate eczema with or without minor infections received a score of 2. Patients with chronic but manageable eczema or recurrent infections or both received a score of 3. Patients with severe eczema and recurrent life-threatening infections received a score of 4. A score of 5 was assigned when patients with eczema and/or frequent infections had developed autoimmune diseases or malignancies.

### Statistical Analysis

Qualitative variables were calculated as median (minimum-maximum), whereas categorical variables were calculated as frequency (percentage).

### Mutation Analysis

Mutation analysis of exons 1 to 12 of the *WAS* gene was performed for 21 of the 23 patients according to the described sequencing technique of Lutskiy et al. [19]. Since there were not sufficient DNA samples for the remaining two patients, their mutation analyses could not be performed.

## Results

### Clinical Features of the Patients

The median age at the onset of symptoms was 15 days (range: 1 day to 7 months). Before admission to our department, 48% (n=11) of the patients were diagnosed with immune thrombocytopenia (ITP), 13% (n=3) with cow’s milk protein allergy (CMPA), and 9% (n=2) with juvenile myelomonocytic leukemia (JMML). The median age at WAS diagnosis was 24 months (1-132 months), and 69% of the patients had a positive family history. The most common complaint was petechia (91%). IVIG (400 mg/kg/3 times weekly) and antimicrobial prophylaxis were given to all. Chronic renal failure (CRF) developed in 3 patients, two of whom had IgA nephropathy and leukocytoclastic vasculitis (LCV) while one had only LCV. In five patients, Epstein-Barr virus (EBV)-associated lymphoproliferation was detected. One patient developed EBV-associated non-Hodgkin’s lymphoma (NHL). Five patients died between 2 and 26 years of age (median: 7 years) due to severe infections (n=2), life-threatening bleeding (n=2), or NHL (n=1). The survival rate without transplantation was 58%, and the oldest patient is 38 years old now. The demographic, clinical, and genetic characteristics and follow-up data of the patients are given in Table 1. WAS scores were mostly 4 or 5 in our patients. The laboratory characteristics and immune work-up results are presented in Table 2.

### Genetic Studies

Two novel mutations were defined in 3 patients (P10, P11, and, P21) from 2 different families. A novel single nucleotide variation mutation in exon 2 (c.273G>C p.Q91H) resulting in a premature stop codon, leading to a shortened transcript, was detected in P10 and P11. In P21 a novel missense mutation was defined in exon 2 c.209G>A that resulted in amino acid changes of  glycine to glutamic acid (p.G70E). Three novel mutations in 4 patients (P12, P17, P18, and P20) were previously reported by the authors [20,21,22]. Known mutations were detected in the remaining 14 patients [3,19,23,24,25,26].

### Data Related to HSCT

Survival was 100% in transplanted patients. HSCT related data are given in Table 3. Eleven patients had received HSCT at a median of 3.5 years (range: 10 months to 9 years). CD34+ stem cells were transfused at a median volume of 6.7x10^6^/kg (3.1-13x10^6^/kg). Either a myeloablative conditioning (MAC) regimen (consisting of a total dose of 16 mg/kg busulfan and 200 mg/kg cyclophosphamide or 16 mg/kg busulfan and 160 mg/m^2^ fludarabine) or a reduced intensity conditioning (RIC) regimen (consisting of treosulfan [>1 year, 42 g/m^2^, <1 year, 36 g/m^2^] and 150 mg/m^2^ fludarabine or 140 mg/m^2^ melphalan and 150 mg/m^2^ fludarabine) was used. The decision of the regimen to be used was made by evaluating the patient’s clinical status, the donor, and the availability of conditioning agents, especially treosulfan. For the prophylaxis of graft-versus-host disease (GvHD), 5 patients received cyclosporine-A (CsA), 5 received CsA and methotrexate, and 1 patient received tacrolimus and mycophenolate mofetil. Anti-thymocyte globulin was added in cases of unrelated donor transplants. Neutrophil and platelet engraftments were achieved on day 14 (range: 10-20) and 27 (range: 17-37), respectively. Acute GvHD (grades I-IV) was observed in 36% of patients. Full donor chimerism was achieved for all patients except P11. Neither autoimmune nor malignant disease developed in any patient during the post-transplant follow-up period of a median of 8.5 years (range: 1-20).

## Discussion

We have evaluated the clinical features and treatment outcomes of the largest series of WAS patients reported from Turkey. Since mucosal bleeding is the most prominent complaint in WAS, misdiagnoses like ITP, JMML, and even CMPA were found to be common among our patients.

ITP is rarely seen in the early years of life [27]. However, according to both our experience and the literature, WAS patients are often diagnosed at first with acute ITP in infancy or chronic ITP in childhood [28,29]. Moreover, since hepatosplenomegaly, leukocytosis, and thrombocytopenia or bloody diarrhea and eczema may be seen in WAS, those presentations can also be misinterpreted as JMML or CMPA [30,31]. In our cohort, almost half of the patients (n=11, 48%) were misdiagnosed with ITP before admission to our department. Likewise, three patients were diagnosed with CMPA and two had a phenotype similar to that of JMML. One had splenectomy before WAS diagnosis. He had severe infections and died of intracranial bleeding (P11). Misdiagnosis may cause diagnostic delay and even fatal consequences, especially in critically ill patients.

Severe thrombocytopenia with low values of mean platelet volume (MPV) (<7 fL) is the most striking finding for the diagnosis of WAS [13,14,15,16]. Normal or high MPVs have been reported in patients with autoimmunity, splenectomy, and repeated thrombocyte transfusions [32,33,34,35]. In our series, the MPV was low in all patients, except for normal values in two patients who had repeated platelet transfusions. 

Patients with autoimmunity who are unresponsive to corticosteroid treatment and/or have not received HSCT have the poorest prognosis [36]. Malignancies, and especially EBV-associated NHL, are reported most frequently in WAS [7,9,15,37]. In our cohort three patients with autoimmune hemolytic anemia who received HSCT did not experience relapses and were cured. One of our patients developed NHL, relapsed, and died, while two with EBV-associated lymphoproliferation were cured following HSCT.

Even in relatives with the same mutation, the severity of clinical findings and survival varied. It was also noteworthy that two patients with an intronic mutation had a JMML-like phenotype. The most striking features of the patients who had novel mutations were very early disease onsets with severe phenotypes (P10, P11, and, P21).

Over the last ten years, age at diagnosis decreased from 48.5 months to 6 months in our cohort. A substantial increase of awareness about WAS and advancements in its diagnosis have contributed to this outcome. Recently, life expectancy was elevated to 20 years, mostly with antimicrobial prophylaxis and IVIG [7,13]. Moreover, HSCT provides excellent outcomes and even cures [12,38]. In a multicenter study evaluating 96 transplanted WAS patients, the overall survival (OS) rate was found to be 97% at 2 years [10]. The most important complication seen after HSCT was autoimmune disease independent of chronic GvHD associated with mixed chimerism. It was observed in 20% of the patients at a median time of +1.5 years and mostly with unrelated donors [10]. Burroughs et al. [39] recently reported the outcome of 129 transplanted WAS patients. Their OS for 5 years was 91% with a median follow-up of 4.5 years. It was shown that HSCT performed in the first 5 years was more successful. They also found that the type of donor and conditioning intensity did not affect OS [39].

In our cohort, HSCT was not available for the patients diagnosed before 2000. They had poor quality of life with several morbidities and high mortality (42%, n=5) due to bleeding, infections, and malignancy. In transplanted patients, however, uneventful OS was 100% in a median of 8.5 years (range: 8 months to 20 years).

In our study, we used either MAC (n=8) or RIC (n=3) regimens. None of the patients had graft failure. A stable mixed chimera developed in only one patient transplanted with a MAC regimen. He has had moderate but clinically insignificant thrombocytopenia for many years [40].

## Conclusion

All male babies with thrombocytopenia should have their MPV measured and should be investigated for family history and the presence of infections and eczema for WAS diagnosis. Furthermore, diagnoses of ITP, leukemia, and CMPA can mask the diagnosis of WAS. Although the survival of WAS patients has increased with supportive treatment recently, patients can die at any time due to bleeding, infections, and malignancy. As HSCT has a 100% success rate in these patients, it should be performed as early as possible after the diagnosis with the most appropriate donor. Gene therapy is also a promising option that has the advantage of avoiding HSCT complications [41].

## Figures and Tables

**Table 1:**
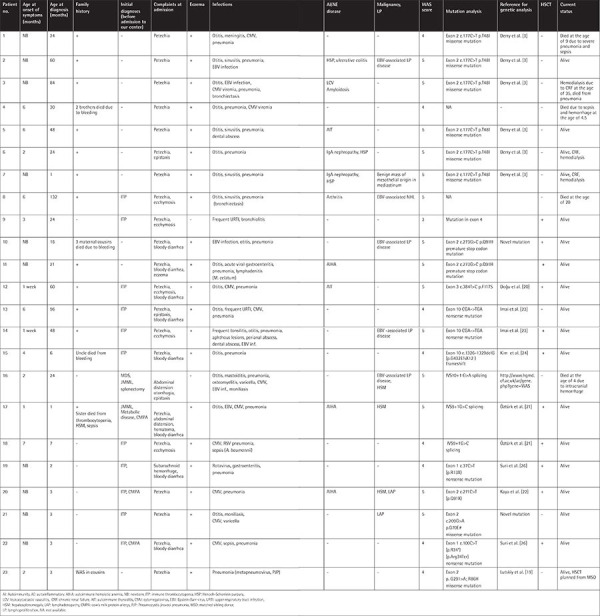
Demographic, clinical, and genetic features and treatments of the patients with outcomes.

**Table 2:**
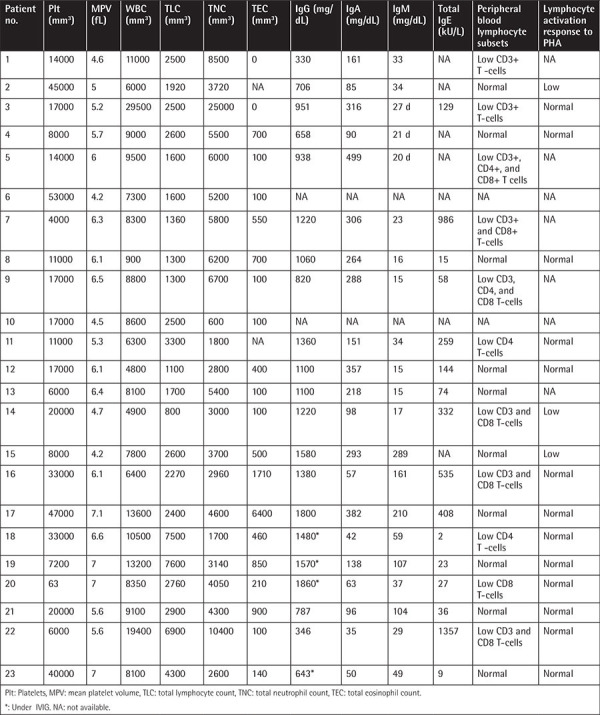
Laboratory features and immune work-up.

**Table 3:**
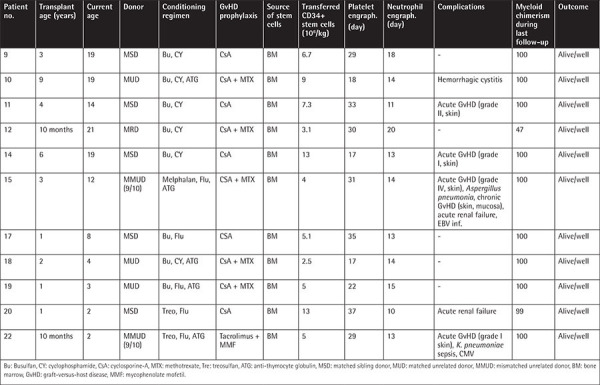
Data of patients treated by HSCT.
